# Multi‐scale nested UNet with transformer for colorectal polyp segmentation

**DOI:** 10.1002/acm2.14351

**Published:** 2024-03-29

**Authors:** Zenan Wang, Zhen Liu, Jianfeng Yu, Yingxin Gao, Ming Liu

**Affiliations:** ^1^ Department of Gastroenterology, Beijing Chaoyang Hospital the Third Clinical Medical College of Capital Medical University Beijing China; ^2^ Hunan Key Laboratory of Nonferrous Resources and Geological Hazard Exploration Changsha China

**Keywords:** colorectal polyp, deep learning, polyp segmentation, transformer

## Abstract

**Background:**

Polyp detection and localization are essential tasks for colonoscopy. U‐shape network based convolutional neural networks have achieved remarkable segmentation performance for biomedical images, but lack of long‐range dependencies modeling limits their receptive fields.

**Purpose:**

Our goal was to develop and test a novel architecture for polyp segmentation, which takes advantage of learning local information with long‐range dependencies modeling.

**Methods:**

A novel architecture combining with multi‐scale nested UNet structure integrated transformer for polyp segmentation was developed. The proposed network takes advantage of both CNN and transformer to extract distinct feature information. The transformer layer is embedded between the encoder and decoder of a U‐shape net to learn explicit global context and long‐range semantic information. To address the challenging of variant polyp sizes, a MSFF unit was proposed to fuse features with multiple resolution.

**Results:**

Four public datasets and one in‐house dataset were used to train and test the model performance. Ablation study was also conducted to verify each component of the model. For dataset Kvasir‐SEG and CVC‐ClinicDB, the proposed model achieved mean dice score of 0.942 and 0.950 respectively, which were more accurate than the other methods. To show the generalization of different methods, we processed two cross dataset validations, the proposed model achieved the highest mean dice score. The results demonstrate that the proposed network has powerful learning and generalization capability, significantly improving segmentation accuracy and outperforming state‐of‐the‐art methods.

**Conclusions:**

The proposed model produced more accurate polyp segmentation than current methods on four different public and one in‐house datasets. Its capability of polyps segmentation in different sizes shows the potential clinical application

## 1 INTRODUCTION

Colorectal cancer (CRC) is one of the leading causes of death around the world. It is reported that a polyp miss detection rate puts patients at high risk of dying from CRC.^1^, ^2^ Accordingly, early detection of colorectal polyps is an essential task of colonoscopy that can reduce the incidence of CRC. In particular, accurate polyp detection is indispensable because every 1% increase in adenoma detection is associated with a 3% decrease in CRC incidence.^3^ Therefore, efforts have been made to improve early detection of polyps. However, it is difficult to accurately detect polyps due to the variation in size, shape, and experience of the colonoscopist. Hence, computer‐aided diagnosis (CAD) technology is aimed at assisting clinicians in medical diagnosis for the early detection of the lesion area. Toward constructing a CAD system, conventional methods and machine learning‐based methods have been proposed.^4^, ^5^, ^6^, ^7^ Compared to conventional methods, automatic segmentation of colonic polyps using a neural network can increase the efficiency of detection and segmentation and reduce the polyp misdetection rate, which is beneficial for the prevention and treatment of CRC.

In recent years, with the development of deep learning, convolutional neural networks (CNNs) have achieved remarkable performance in the field of segmentation of natural and medical images. The UNet^6^ with symmetric encoder‐decoder paradigm and a skip‐connection‐based network has shown excellent segmentation performance for biomedical images. The encoder extracts features by successive downsampling, and the decoder gradually aggregates the feature output from the encoder via skip connection with features upsampled from the previous decoder layer to the input resolution. The high‐resolution features are reused with skip connections at different resolutions to recover the spatial information lost by downsampling from the high‐resolution representations, which benefits the network of combining low‐resolution features and high‐resolution features, resulting in better segmentation performance. The success of encoder‐decoder‐based networks is mainly due to their skip connections, which enable the propagation of deep, semantically significant, and dense feature maps from the encoder network to the decoder subnetworks. However, such a design is constrained by the optimal depth and design of skip connections. Based on the U‐shaped network, several novel models such as ResUNet++,^7^ Attention UNet,^8^ DenseUNet,^9^ R2UNet^10^ been proposed specifically for medical image segmentation and achieve remarkable performance. Despite the good performance, this kind of approach is still unable to explore sufficient information from multiple scales. Furthermore, UNet++^11^ has intensified the connections by introducing nested and dense skip connections to reduce the semantic gap between the encoder and the decoder. Moreover, the CNN‐based approaches^12^, ^13^, ^14^ are limited in learning global information and long‐range dependencies.^15^, ^16^

Recently, inspired by the great success of transformer in the field of natural language processing (NLP), researchers have introduced a transformer to the field of computer vision. Vision Transformer (ViT)^17^ is the first work that introduced a transformer for image recognition, which achieves comparable performance with other CNN‐based methods by pretraining on large datasets. Furthermore, to reduce the computational complexity, a hierarchical Swin Transformer with Window‐based MSA (W‐MSA) and Shifted Window‐based MSA (SW‐MSA) is developed, which achieves the state‐of‐the‐art (SOTA) performance on various computer vision tasks including image classification, detection, and segmentation. TransUNet^15^ employs the transformer as a bridge to connect the encoder and decoder in a U‐shaped network to model long‐range dependencies. DS‐TransUNet^18^ uses two Swin Transformer branches of the encoder that learns feature representations of different scales. TransFuse^19^ tries to fuse the features extracted by ViT and CNNs. The success of these models shows the great potential of the transformer in medical image segmentation.

In this work, we propose a novel architecture for polyp segmentation, a multiscale nested UNet structure with an integrated transformer. The proposed network takes advantage of both the CNN and transformer to extract distinct feature information. To be specific, low‐level flattened features with positional embedding are passed to the transformer as input to learn explicit global context and long‐range semantic information while CNNs using Resnet as the backbone to extract local information within a receptive field. Moreover, to address the variant sizes of polyp, a multiscale feature fusion (MSFF) unit is proposed to fuse features with multiple resolutions.

The main contributions of this work are as follows:

1. The proposed model combines the CNN with transformer structure, which allows the model to learn both local and global context information.
2. A MSFF is proposed, which facilitates the model potential to capture polyps of various sizes and shapes via MSFF.
3. The proposed method has been extensively validated and compared to multiple methods on four public datasets: Kvasir‐SEG,^20^ CVC‐ClinicDB,^21^ CVC‐ColonDB,^22^ ETIS‐Larib,^23^ as well as an in‐house dataset.

## 2 MATERIALS AND METHODS

**FIGURE 1:**
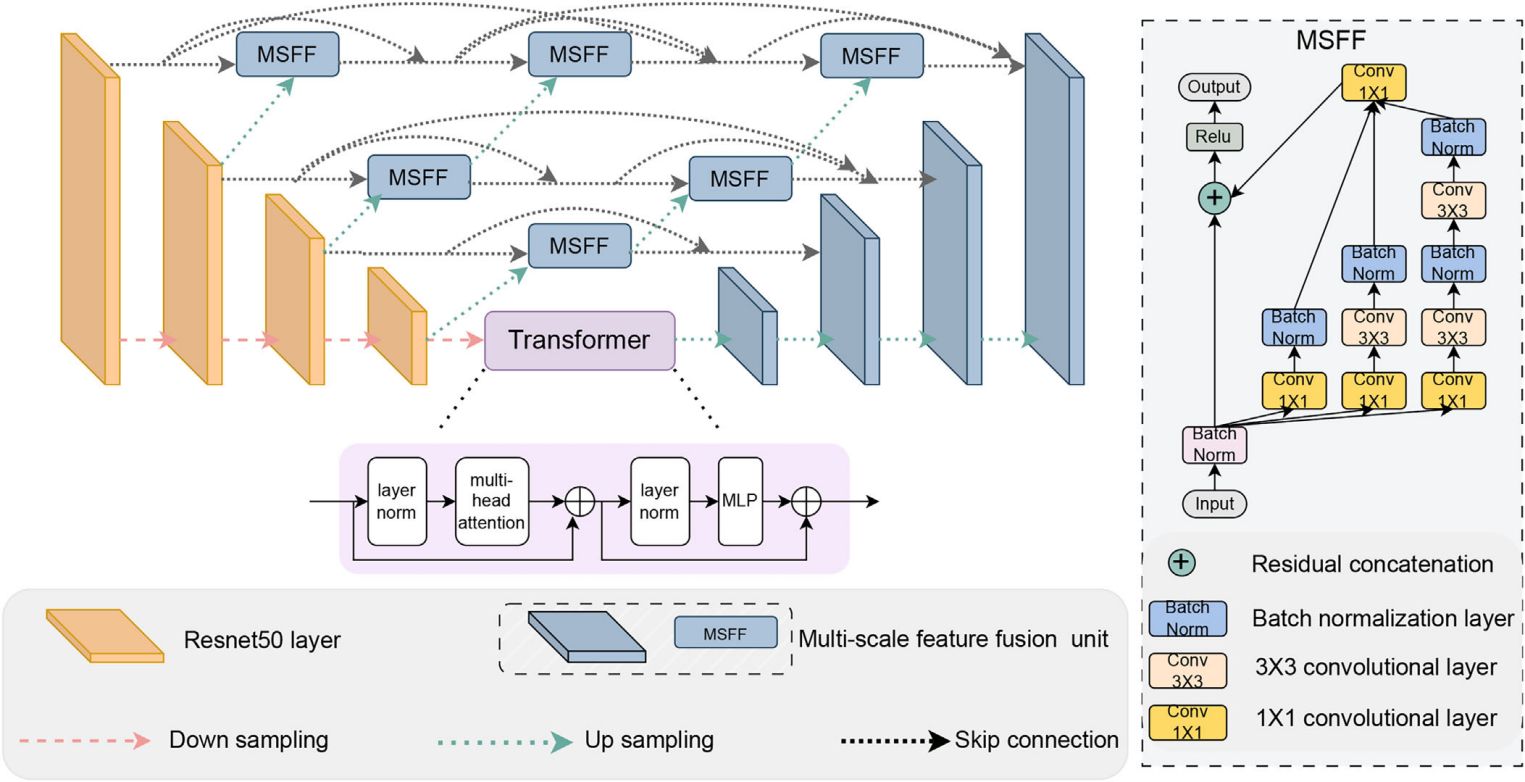
The proposed model architecture. The proposed model is comprised of three parts: encoder, transformer, and decoder. ResnetV2‐50 is used as the encoder to extract features from the input image. Extracted features are passed to the decoder through a densely connected skip connection. Features of the last encoder stage are flattened and fed into the transformer for long‐range dependency modeling. MSFF as the basic component is used in the intermediate nodes and decoder stages to fuse features from multireception with concatenation. MSFF, multiscale feature fusion.

In this section, the components of the proposed model architecture are presented. The overall structure of the proposed model is presented in detail and shown in Figure 1. We introduced the MSFF unit to capture multiscale features in the UNet++ structure and to capture the long‐range dependencies of the features. ViT serves as a bridge to connect encoders and decoders.

### 2.1 U‐shape segmentation net

We implement nested UNet as the basic structure and use ResnetV2‐50^24^ as the backbone for encoder feature extraction. The features are extracted through the four stages forming features with different resolutions and then are merged with the decoder through a skip connection which is densely connected to reduce the semantic gap between the encoder and the decoder. The deep residual unit makes the deep network easy to train, and the skip connection within the network helps to propagate gradient information, which improves the neural network design by reducing the parameters with comparable or increasing performance for the semantic segmentation task.

To be specific, the encoder consists of four blocks, one stem and three residual blocks, consisting of residual units. Each residual unit is a combination of group normalization (GN), Rectified Linear Unit (ReLU) activation, convolutions, and identity mapping. The identity mapping connects the input and output which makes the network go deeper. In each residual block, 1×1 and 3×3 are used as the kernel size, and a strided convolutional layer is used to reduce the spatial dimension of the feature maps by half instead of max pooling. The outputs of each residual block are used to format the nested UNet, and the output of the last encoder block is flattened and passed to the transformer to learn global information and long‐range dependencies.

### 2.2 Multi‐scale feature fusion (MSFF) unit

The variety of geometric shapes and sizes makes it challenging for polyp detection and segmentation. To address this issue, we introduce a MSFF unit with a modified Inception‐Resnet module to fuse features with a combination of different sizes of convolutional filters to obtain different scales of receptive fields, as shown in Figure 1. In the decoder stage and intermediate node, the up‐sampled features and the skip connection are concatenated as the input to the MSFF unit. The motivation is that the features with a higher resolution from shallow layers contain boundary information while features with a lower resolution from deeper layers contain more contextual and semantic information. To merge features from different resolutions, we aim to have the feature contain both contextual‐sufficient and boundary information for segmentation.

To be specific, the MSFF unit contains two parts: convolutional block (CB) and Inception‐Resnet. CB captures the information with a 3×3 convolutional layer followed by batch normlization (BN) and ReLU activation. Inception‐Resnet is composed of one identity mapping and three convolutional branches $b_{1},b_{2},b_{3}$. The convolutional layer of Inception‐Resnet is followed by a BN layer. Identity mapping is used to preserve the original feature map information to prevent gradient explosion with a scaling α for residual scaling. Finally, a ReLU layer is used for activation and output. MSFF unit can be formulated as follows:

(1) $$\begin{array}{ccc} x^{\ell } & = & CB\left(x^{\ell }\right) \end{array}$$

(2) $$\begin{array}{ccc} x^{\ell +1} & = & ReLU\left(x^{\ell }+\alpha \times \left[x_{b1}^{\ell },x_{b2}^{\ell },x_{b3}^{\ell }\right]\right) \end{array}$$

where, [·] is the concatenation operation. $x^{\ell },x^{\ell +1}$ denotes input and output of ℓ+1‐th layers. $x_{bi}^{\ell },i\in \left[1,2,3\right]$ stands for the output of three branches of ℓ‐th layers of the Inception‐Resnet. α∈[0,1] denotes the scale factor. We set α=1 in all the experiments.

### 2.3 Vision transformer layer

The convolutional operation of CNN constrains the receptive field of CNN by the kernel size. A larger receptive field could be achieved by the stacking of multiple CNN layers with a small kernel size or using a large kernel size, which, however, increases the parameters. To achieve modeling the long‐range dependencies between pixels, we propose to use a multihead self‐attention (MSA) layer positioned between the encoder and the decoder.

To achieve this, we first formulate the last output of the encoder (H×W×C) into a sequence of 2D patches $\left\{x_{p}^{i}\in R^{P^{2}\times C}i=1,..,N\right\}$, where H×W is the size of the input feature, C is the number of channels, (P,P) is the patch size, and $N=\frac{HW}{P^{2}}$ is the number of feature patches. The feature patches are flattened to the 1D vector with a trainable projection to features of Q,V, and K. To obtain ultimate spatial information, a learnable 1D position embedding is added to the patch embedding. The transformer layer consists of L layers of MSA and multilayer perceptron (MLP) blocks. MSA concatenates multiple SAs, shown in Equation (3)

(3) $$\begin{array}{ccc} SA(Q,K,V) & = & Softmax\left(\frac{QK^{T}}{\sqrt{D}}\right)v \end{array}$$

(4) $$\begin{array}{ccc} MSA(Q,K,V) & = & Concate(\left[H_{1},\text{…},H_{h}\right]) \end{array}$$

Where h denotes the total number of heads and $Q,K,V\in R^{N\times N,D}$ are query,key and value, respectively. The Q attends to all the locations of the features, then increases the receptive field limited by CNN.

### 2.4 Loss function

We implement a combination of binary cross‐entropy loss $L_{bce}$ as defined in Equation (5) and dice loss $L_{dice}$, where y is the ground truth and $\hat{y}$ is the predicted map. The loss function is defined as follow:

(5) $$\begin{array}{ccc} L_{bce} & = & \left(y-1\right)log\left(1-\hat{y}\right)-ylog\hat{y} \end{array}$$

(6) $$\begin{array}{ccc} L_{dice} & = & 1-\frac{2y\hat{y}+1}{y+\hat{y}+1} \end{array}$$

(7) $$\begin{array}{ccc} L_{decoder} & = & \lambda_{1}L_{bce}+\lambda_{2}L_{dice} \end{array}$$

The sum of the two loss functions is used for gradient minimization between the predicted maps and the labels.

### 2.5 Experimental setup

#### 2.5.1 Implementation details

The proposed method was implemented with the PyTorch library.^25^ All experiments were conducted on NVIDIA V100 Tensor Core GPU with 32 GB GPU memory. The model was trained for a total of 200 epochs using the Stochastic Gradient Descent (SGD) with momentum as 0.9 and weight decay as $1e^{-4}$. The initial learning rate was set to $5e^{-3}$, and the polynomial learning decay rate schedule was used. $\lambda_{1}$ and $\lambda_{2}$ in loss function were set to 0.5. The ImageNet pretrained weights of ViT^17^ were loaded for the transformer which contains 12 layers before training; other layers were trained from scratch.

Several data augmentation strategies were employed in this study, including random rotation between 20 degrees, random horizontal and vertical flip, and random Gaussian blur. All the images were resized to 224×224 to reduce computational complexity and improve training efficiency.

#### 2.5.2 Dataset

The public datasets of CVC‐ClinicDB,^21^ CVC‐ColonDB,^22^ ETIS‐Larib PolypDB,^23^ and Kvasir‐SEG^20^ and a private in‐house dataset are used to evaluate our model. CVC‐ColonDB contains 380 images with associated polyp masks obtained from 13 polyp video sequences from 13 patients. CVC‐ClinicDB contains 612 images with associated polyps, background (mucosa and lumen in this case), and segmentation masks obtained from 31 polyp video sequences from 23 patients.

ETIS‐Larib is a database of images extracted from colonoscopy videos. These images contain multiple samples of polyps with a mask corresponding to the area covered by polyps.

Kvasir‐SEG contains 1000 images and their corresponding ground truth masks annotated byexperienced endoscopists.

**FIGURE 2:**
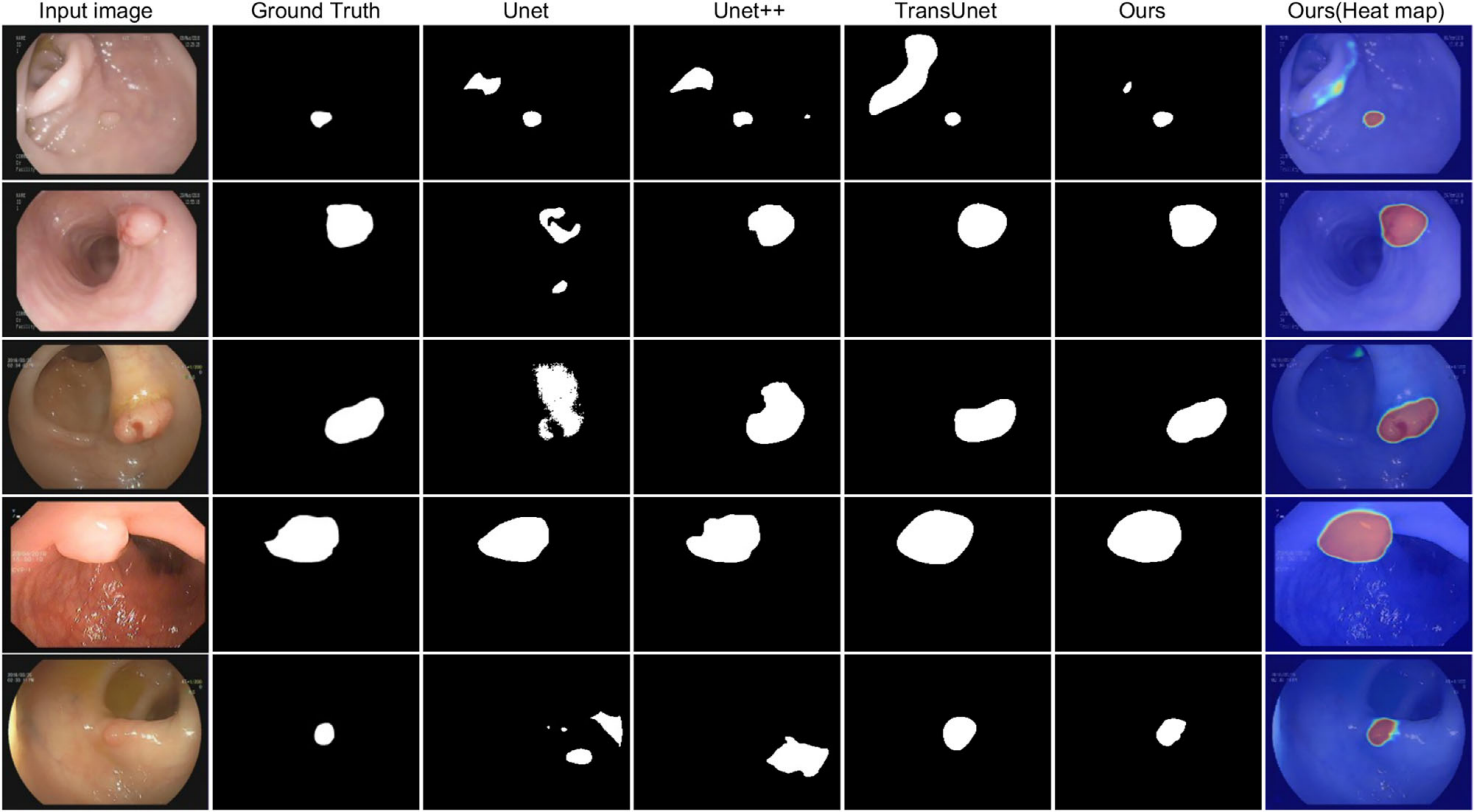
Segmentation results on in‐house dataset. In particular, the first row demonstrated more precise results of our model than others. The corresponding heat map proved this further.

The in‐house dataset was gathered from the Beijing Chaoyang Hospital, China, which contains 229 images of 65 patients. Each image has at least one polyp in it. Four experienced endoscopists labeled the data and cross‐verified with a formal diagnostic report. This data is fully approved. Details can be seen in Table 1 and Figure 2.

**TABLE 1:** The colorectal polyp datasets used in this study.

Dataset	Images	Resolution	Ground Truth	Year	Availability
CVC‐ColonDB^22^	380	574×500	Binary mask	2012	Public
ETIS‐Larib PolypDB^23^	196	1225×966	Binary mask	2014	Public
CVC‐ClinicDB^21^	612	384 × 288	Binary mask	2015	Public
Kvasir‐SEG^20^	1000	Variable	Binary mask	2020	Public
In‐house	229	768 × 576	Binary mask	2022	Private

#### 2.5.3 Evaluation metrics

To compare our proposed model with other methods, four standard evaluation metrics for medical image segmentation were used including precision, recall, mean intersection over union (mIoU), and mean dice coefficient (mDice). Dice and IoU are the two most commonly used metrics for medical image segmentation.^6^, ^7^, ^11^, ^26^, ^27^, ^28^ Dice is used to compare the similarity between the predicted segmentation results and the original ground truth mask. IoU is used to compare the overlap between the predicted mask and the ground truth. The details of different performance metrics are listed in Table 2.

**TABLE 2:** Performance metrics for polyp detection.

Metric	Abbreviation	Calculation
Precision	Prec	$Prec=\frac{TP}{TP+FP}$
Recall	Rec	$Rec=\frac{TP}{TP+FN}$
Dice	Dice	$Dice=\frac{2TP}{2TP+FP+FN}$
IoU	IoU	$IoU=\frac{TP}{TP+FP+FN}$

*Note* TP, TN, FP, and FN stand for true positive, true negative, false positive, and false negative, respectively.

## 3 RESULTS AND DISCUSSION

In this section, we present the comparison results of the proposed model to other methods on four public datasets and one in‐house dataset.

### 3.1 Comparison on Kvasir‐SEG

Firstly, we evaluated the performance of our proposed method on the Kvasir‐SEG dataset. With the same split strategy in FANet,^27^ 880 images were used for training and 120 images were used for testing. The results listed in Table 3 showed that our model outperformed HarDNet‐MSEG^28^ and FANet^27^ by a large margin in terms of mDice, mIoU, recall, and precision and slightly outperformed MSRF‐NET^29^ in terms of mDice, mIoU, and recall. In particular, our model achieved a mDice of 0.942, mIoU of 0.894, recall of 0.939, and precision of 0.950, which achieved 2.28% improvement on mDice and 0.3% improvement on mIoU as compared with MSRF‐NET.^29^

**TABLE 3:** Result comparison on Kvasir‐SEG.

Method	mDice	mIoU	Recall	Precision
UNet^6^	0.597	0.471	0.617	0.672
UNet++^11^	0.747	0.631	0.686	0.887
ResUNet++^7^	0.714	0.613	0.742	0.784
PraNet^26^	0.899	0.840	−	−
DoubleUNet^30^	0.813	0.733	0.840	0.861
FANet^27^	0.880	0.815	0.906	0.901
HarDNet‐MSEG^28^	0.904	0.848	0.923	0.907
MSRF‐NET^29^	0.921	0.891	0.919	**0.966**
Ours	**0.942**	**0.894**	**0.939**	0.950

*Note* The best results are highlighted in bold. “—” means results are not available.

### 3.2 Comparison on CVC‐ClinicDB

For CVC‐ClinicDB, 550 images were used to train the model and 62 images were used for testing.^7^ Comparison results are listed in Table 4, which showed that our proposed model outperformed all the other methods in terms of mDice and recall. Our precision was still competitive with the best performing DoubleUNet.^30^ To be specific, our model achieved mDice of 0.950, mIoU of 0.901, recall of 0.957, and precision of 0.940 and outperformed the previous SOTA method MSRF‐NET^29^ in terms of mDice and recall.

**TABLE 4:** Result comparison on CVC‐ClinicDB.

Method	mDice	mIoU	Recall	Precision
UNet^6^	0.823	0.755	−	−
UNet++^11^	0.794	0.729	−	−
ResUNet++^7^	0.795	0.796	0.702	0.878
PraNet^26^	0.899	0.849	−	−
DoubleUNet^30^	0.923	0.861	0.845	**0.959**
FANet^27^	0.935	0.893	0.933	0.940
MSRF‐NET^29^	0.942	**0.904**	0.956	0.942
Ours	**0.950**	0.901	**0.957**	0.940

*Note* The best results are highlighted in bold. “—” means results are not available.

### 3.3 Cross dataset validation on public datasets

We designed two cross‐dataset validation experiments. Firstly, the training set consists of 900 images from Kvasir‐SEG and 550 images from CVC‐Clinic DB, the same as ref. 26. The testing set from the other unseen datasets is 100 images from Kvasir‐SEG, 62 images from CVC‐ClinicDB, 380 images from CVC‐ColonDB, and 196 images from ETIS‐Larib. The purpose of this experiment is to show the generalization of the different methods. Results are listed in Table 5, which demonstrated that our model outperformed all the current methods with exception of the ETIS‐Larib dataset. Our model achieved a mDice of 0.940 on Kvasir‐SEG, mDice of 0.944 on CVC‐ClinicDB, mDice of 0.785 on CVC‐ColonDB, and mDice of 0.701 of ETIS‐Larib.

**TABLE 5:** Cross‐evaluation of mDice score on four datasets.

Method	Kvasir‐SEG	CVC‐ClinicDB	CVC‐ColonDB	ETIS‐Larib
UNet^6^	0.818	0.823	0.512	0.710
UNet++^11^	0.821	0.794	0.483	0.707
PraNet^26^	0.898	0.899	0.709	0.628
HarDNet‐MSEG^28^	0.912	0.932	0.731	0.677
TransFuse‐S^19^	0.918	0.918	0.773	**0.733**
Ours	**0.940**	**0.944**	**0.785**	0.701

*Note* Training on Kvasir‐SEG and CVC‐ClinicDB. Test on Kvasir‐SEG, CVC‐ClinicDB, CVC‐ColonDB, and ETIS‐Larib. The best results are highlighted in bold.

Secondly, we trained the model on Kvasir‐SEG and CVC‐ClinicDB, that is, training on one dataset and testing on the other one, the same as in ref. 29. Results are listed in Table 6. Training on Kvasir‐SEG gave the model a mDice of 0.855 on CVC‐ClinicDB and 0.654 on ETIS‐Larib. Oppositely, training on CVC‐ClinicDB let our model achieve a mDice of 0.853 on Kvasir‐SEG and 0.618 on ETIS‐Larib. The two cross‐validation experiments demonstrated performance and generalization of our model.

**TABLE 6:** Cross‐evaluation results of mDice score on CVC‐ClinicDB, Kvasir‐SEG, and ETIS‐Larib.

	Kvasir‐SEG		CVC‐ClinicDB	
Method	CVC‐ClinicDB	ETIS‐Larib	Kvasir‐SEG	ETIS‐Larib
UNet^6^	0.750	0.602	0.668	0.575
ResUNet++^7^	0.671	0.400	0.721	0.397
DoubleUNet^30^	0.753	0.644	0.676	0.612
PraNet^26^	0.722	−	0.729	−
MSRF‐NET^29^	0.7921	−	0.7575	−
Ours	**0.855**	**0.654**	**0.853**	**0.618**

*Note* The best results are highlighted in bold. “—” means results are not available.

### 3.4 Results of in‐house dataset

**FIGURE 3:**
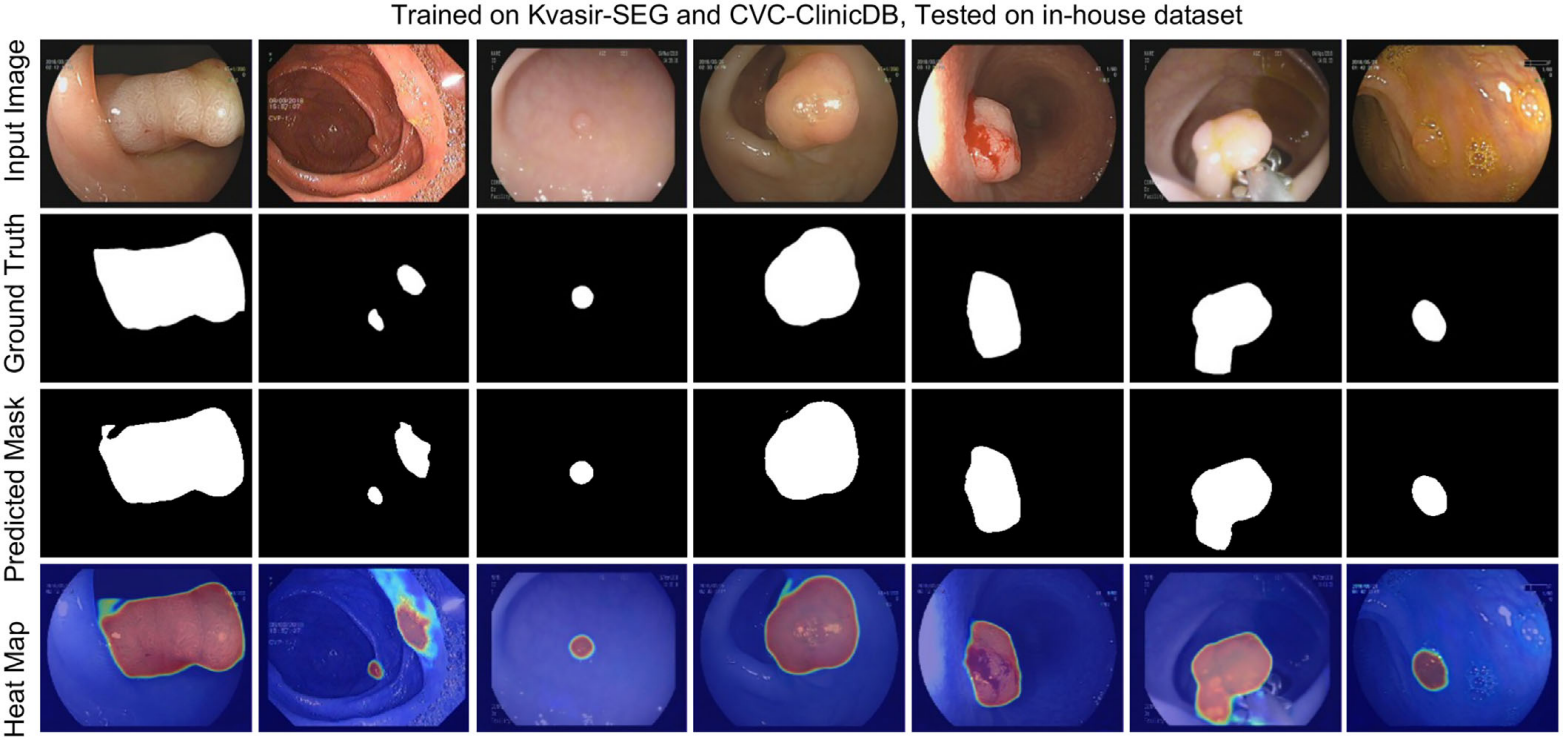
Segmentation results on in‐house dataset. Training on Kvasir‐SEG and CVC‐ClinicDB; test on in‐house dataset.

We evaluated our proposed model on the private dataset. Firstly, we straightforward evaluated the model by the strategy presented in Section 3.3; that is, the model was trained with Kvasir‐SEG, CVC‐ClinicDB, and the mix of these two datasets separately and tested on the entire in‐house dataset. Evaluation results are listed in Table 7. To be specific, the model trained on CVC‐ClinicDB resulted in a mDice of 0.789 when tested on the in‐house dataset. Accordingly, training on Kvasir‐SEG gave a mDice of 0.841, while training on the mix of these two datasets led to a mDice of 0.850. These results showed that our model had strong scalability and stable performance on the unseen data. Segmentation results can be seen in Figure 3. Furthermore, we split the dataset with 90: 10 for training and testing. We evaluated UNet,^6^ UNet++^11^ and TransUNet^15^ on the in‐house dataset separately; comparison results are listed in Table 8. Our model achieved the best results of mDice, mIoU, recall, and precision of 0.910, 0.839, 0.905, and 0.925, respectively. Figure 2 shows the segmentation results and corresponding heat map.

**TABLE 7:** Cross‐evaluation results on in‐house dataset.

	CVC‐ClinicDB	Kvasir‐SEG	Kvasir‐SEG & CVC‐ClinicDB
Ours	0.789	0.841	0.850

*Note* Training on CVC‐ClinicDB, Kvasir‐SEG, and mix of them; test on in‐house dataset.

**TABLE 8:** Result comparison on in‐house dataset.

**Method**	mDice	mIoU	Recall	Precision
UNet^6^	0.488	0.406	0.500	0.585
UNet++^11^	0.701	0.600	0.729	0.741
TransUnet^15^	0.848	0.760	0.896	0.847
Ours	**0.910**	**0.839**	**0.905**	**0.925**

*Note* The best results are highlighted in bold.

### 3.5 Ablation study

To evaluate the improvement of components of MSFF and the transformer, we performed ablation tests on the CVC‐ClinicDB dataset. The training set and test set were the same as in Section 3.2. Separate networks were trained from scratch with the following settings: (a) backbone (nested UNet); (b) backbone with MSFF; (c) backbone with transformer; (d) the proposed model. The ImageNet pretrained weights of ViT^17^ were loaded to initial corresponding layers. The results are shown in Table 9. The backbone network led to a mDice of 0.789. Furthermore, we added MSFF to the backbone, which brought a significant improvement and gave a mDice of 0.89. To further investigate the importance of the transformer, we added transformer layers to the backbone to achieve a higher mDice of 0.939. Finally, we added MSFF and the transformer layer, which obtained the best results in terms of mDice, mIoU, recall, and precision. These results clearly indicated that MSFF and the transformer increased accuracy and achieved the best results.

**TABLE 9:** Ablation study on CVC‐ClinicDB.

Method	mDice	mIoU	Recall	Precision
Backbone^11^	0.847	0.751	0.805	0.921
Prop without Transformer	0.892	0.817	0.874	0.933
Prop without MSFF	0.939	0.887	0.940	0.939
Proposed Model	**0.950**	**0.901**	**0.957**	**0.940**

*Note* The best results are highlighted in bold.

### 3.6 Discussion

**FIGURE 4:**
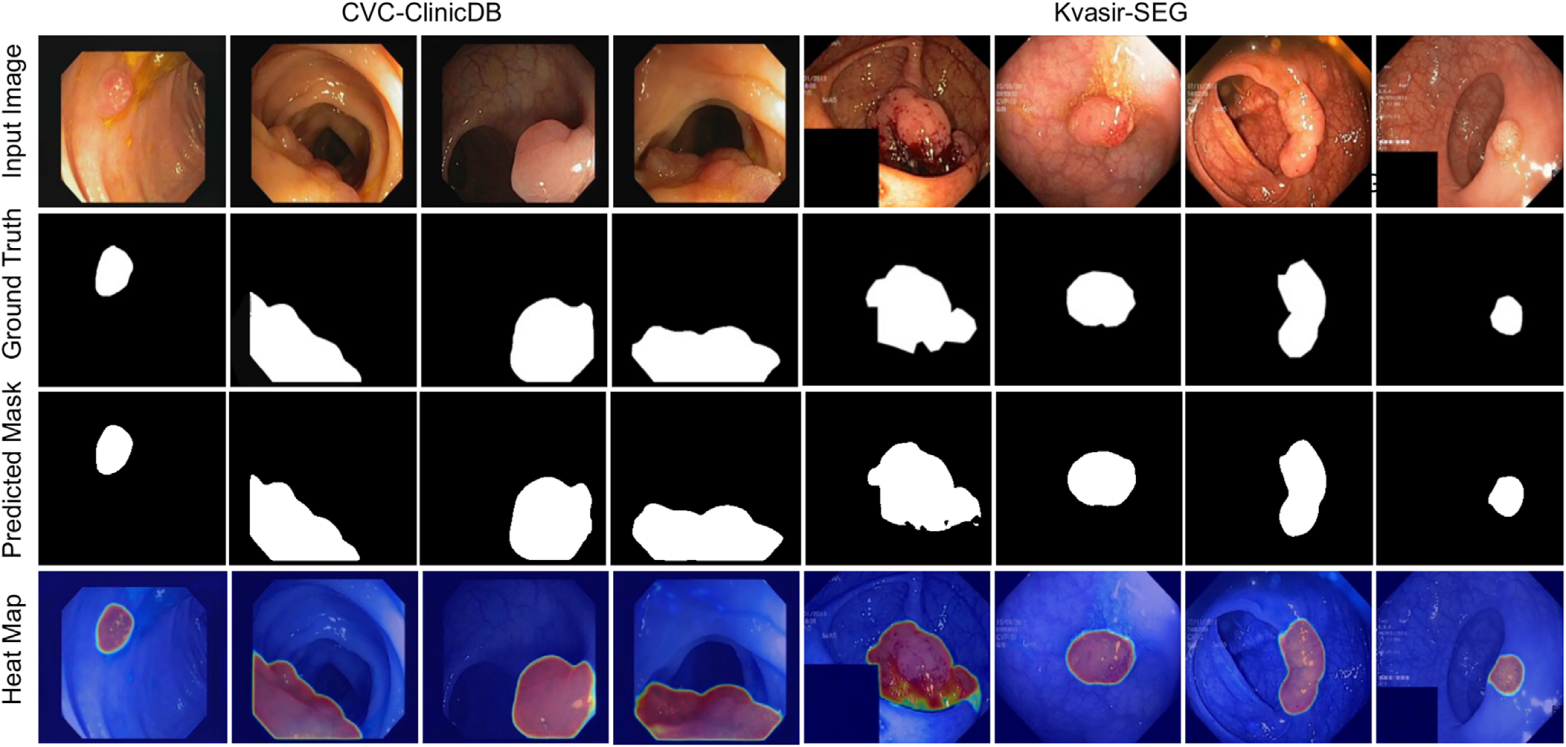
Segmentation results on datasets of CVC‐ClinicDB and Kvasir‐SEG. The first row is the input image, the second row is the corresponding ground truth, the third row is the prediction of the model, and the last row is the heat map of the last layer.

In this work, we developed a new architecture combined with the nested UNet and transformer with multiple‐resolution fusion for polyp segmentation. Our proposed model outperformed SOTA methods on three public datasets. The proposed model was significantly more accurate than the pure CNN‐based model (Table 4, 3, 5, 6). The proposed model also outperformed the other transformer‐based methods such as TransFuse.^19^ Furthermore, the segmentation results in Figures 4 and 3 demonstrated that the proposed model achieved better performance than the other methods on a variety of polyps.

Transformer shows the superiority of modeling long‐range dependencies, and transformer‐based methodologies have been studied for medical image segmentation.^31^ But the lack of capability of capturing context information restricts the power of the transformer.^31^ Studies on leverage ViT and CNN as encoders both take images as inputs. TransUNet^15^ uses the transformer as the bridge to connect the encoder and decoder. But the UNet structure suffers from a semantic gap,^29^ and lacks multiscale feature representation. To this end, we proposed a hybrid CNN‐Transformer feature extraction encoder to capture local and long‐range dependencies. The skip connection densely concatenated features from different resolutions. The nodes between the encoder and the decoder employed a MSFF block with a modified Inception‐Resnet unit to capture local information with different scales. Additionally, the MSFF units permitted the proposed model to effectively capture the variability in size, shape, and structure of the region of interest. The decoder is aggregated with nodes at the same scale. All the features were fused with the proposed MSFF unit. Thus, the aggregated decoder layer enhanced the multiscale feature capture ability, which led to a more precise localization. From the single‐dataset experiments, we can observe that the proposed model achieved the highest dice score of 0.942 and mIoU of 0.894 on the Kvasir‐SEG dataset (Table 3). Similarly, we also achieved the highest dice score of 0.950 and recall of 0.957 on the CVC‐ClinicDB dataset (Table 4), and the second highest mIoU score of 0.901 and a relatively high precision score of 0.940.

In practical clinical applications, the models that are able to generalize across multicenter datasets are more reliable. For this purpose, we conducted the cross‐dataset validation experiments. Our proposed model achieved the highest mDice of 0.94 on Kvasir‐SEG, 0.944 on CVC‐ClinicDB, 0.785 on CVC‐ColonDB, and competitive mDice for ETIS‐Larib when trained on Kvasir‐SEG and CVC‐ClinicDB (see Table 5).We also achieved the highest mDice score of 0.855 on CVC‐ClinicDB and 0.654 on ETIS‐Larib when trained on Kvasir‐SEG. While training on CVC‐ClinicDB, we got a mDice score of 0.853 when tested on Kvasir‐SEG and 0.618 when tested on ETIS‐Larib and outperformed all the comparison methods (see Table 6). The experiment results showed that our proposed model is much more generalizable than other SOTA methods. This could be due to the model structure that captures both local and global long‐range dependencies and MSFF. Finally, the in‐house private dataset also showed the scalability and stabilization of the proposed model.

We performed an ablation study (Table 9) to demonstrate that the combination of MSFF and the transformer is important for boosting the segmentation performance. Without using the transformer layer, the mDice dropped from 0.950 to 0.892 indicating the effectiveness of long‐range dependency modeling. We also verified the MSFF unit, the mDice dropped from 0.948 to 0.939 when the MSFF unit was disabled, and similar findings have been shown in ref. 32.

Medical images contain complex organ anatomies, showing strong global correlations. Accurately segmenting and comprehending these interconnected components is crucial. The proposed approach takes the long‐range dependency modeling ability to achieve this. It can be applied to diverse medical image segmentation tasks, such as MRI and CT images, and encourages further research in medical image analysis.

This work has a few limitations that need to be addressed. Firstly, the proposed model failed to achieve the best results on ETIS‐Larib with cross‐evaluation, as shown in Table 5. The primary reason for this could be the distribution gap between ETIS‐Larib and other datasets. Most of the polyps in this dataset are relatively small, flat, and distant, which makes localization and segmentation difficult. Moreover, the images are downsampled to a small size for model consideration, making it even more challenging to detect small polyps. Hence, it would be beneficial to investigate more efficient data augmentation techniques to overcome this challenge and improve the model's performance. Furthermore, MSRF‐NET has achieved higher precision than our model in Table 3. This could be due to the effective features fusion block, which captures the variability in the structure of the region of interest efficiently. Additionally, the residual structure allows MSRF‐NET^29^ to cater to the demands of detecting small polyps in the image. In addition, We conducted an internal test using in‐house data of 229 images from 65 patients with a range of polyps. However, the number is comparatively small due to difficulty in collection and labeling compared to other public datasets. Therefore, we plan to collect more in‐house data in the future work to enhance the model's training performance. Moreover, real‐time detection is essential for clinical use, whereas the current model was evaluated only on still images. Hence, we plan to gather and label images and videos from several institutions to meet the actual needs of the clinical environment.

## 4 CONCLUSION

In this paper, we proposed a new architecture for polyp segmentation that used both CNN and transformers as encoders to capture local information and long‐range dependencies. The experiments on different datasets including four public datasets and one in‐house dataset have shown that our proposed model outperformed the SOTA methods.

## CONFLICT OF INTEREST STATEMENT

The authors declare no competing interests.
